# Exploring different objectives in non-inferiority trials

**DOI:** 10.1136/bmj-2023-078000

**Published:** 2024-06-17

**Authors:** Conor D Tweed, Matteo Quartagno, Michelle N Clements, Rebecca M Turner, Andrew J Nunn, David T Dunn, Ian R White, Andrew J Copas

**Affiliations:** 1 Medical Research Council (MRC) Clinical Trials Unit at University College London (UCL), London WC1V 6LJ, UK

## Abstract

Non-inferiority trials compare the efficacy of a new treatment with an existing one where the new treatment is expected to have broadly similar efficacy to the existing treatment, but where other benefits might make the new treatment desirable. These trials might aim to demonstrate that a new treatment is either an alternative to, or a replacement for, the current treatment. In this article, how treatment comparisons can be based only on efficacy, or on both efficacy and other benefits, is explained, and guidance on how to choose the correct objective for a trial is given. This choice should influence the design of the trial (eg, choosing the non-inferiority margin and secondary outcomes), analysis, and reporting of the trial. Most non-inferiority trials aim to show only that a new treatment is an alternative to the standard of care. Being more transparent about the trial objective, however, could mean that more trials are conducted with an emphasis on the risk-benefit trade-off for a new treatment and generate more clinically meaningful trial results with a greater effect on practice.

Non-inferiority clinical trials conventionally try to show that a new treatment is not appreciably worse than an existing treatment (as the control arm) by a specific amount, known as the non-inferiority margin.^1^ Non-inferiority trials have become more common because the emphasis is on finding new treatments that overcome drawbacks, such as side effects or length of treatment, that could make these new treatments attractive to clinicians managing patients. The additional benefits are frequently not directly assessed as part of the trial,^2^ however, and are more commonly implicit rather than clearly articulated.

Not considering these additional benefits as part of designing an adequately powered trial has two related risks. Firstly, a new intervention with substantial additional benefits that only just fails to achieve conventional non-inferiority compared with the standard of care could be dismissed by policy makers without any consideration of these benefits and how they would translate into real world use. Secondly, when a trial is powered to detect non-inferior efficacy only, formal comparison of the side effects and benefits is not possible, and a difficult-to-tolerate standard of care could remain a recommendation alongside a non-inferior new intervention with a superior side effect profile.

We believe that two potential objectives exist when conducting a non-inferiority trial: evaluation of a new intervention either as an alternative option or as a replacement for the existing treatment. In this article, we provide guidance on how to choose the correct objective for a trial, and how this choice should influence the design, analysis, and reporting of the trial. We also consider the requirements for licensing if this is sought. Lastly, we describe how to use a risk-benefit analysis to assess a new intervention as a replacement for an existing treatment by accounting for additional benefits to declare that the new intervention with non-inferior efficacy is superior overall to the standard of care.

**Summary points**

Non-inferiority trials could be considered as assessing a new treatment either as an alternative option for management or as a replacement for the current treatment
The two objectives can influence the design of a trial (eg, choosing the non-inferiority margin and secondary outcomes), and lead to different approaches to analysis and different requirements for reporting of a trial
Trials assessing a new treatment as a replacement for the current treatment would focus on both efficacy and the magnitude of the additional benefits
Being more transparent about the trial objective should mean more trials are conducted with an emphasis on the risk-benefit trade-off for a new treatment, which should also generate more clinically meaningful trial results

## Objectives in non-inferiority trials

Two reasons or objectives for performing non-inferiority trials exist, which are generally implied in the design of the trial but not formally articulated: assessing a new intervention as an alternative option or as a replacement for an existing treatment. The alternative option is the more common approach. Table 1 summarises the considerations for each objective.

**Table 1:** Considerations in trial design, analysis, and reporting according to proposed objective of alternative option or replacement

Scope	Trial objective	
	Alternative option (efficacy only)	Replacement (risk-benefit trade-off)
**Design considerations**		
Choice of non-inferiority margin	Based on efficacy, and derived from expert opinion or maintained treatment effect compared with placebo, or both	Based on magnitude of other benefits with decision analysis or judgment of panel of patients and clinicians
Choice of outcomes	Primary: efficacy	Primary: efficacy Secondary: outcomes that reflect other benefits or harms
Sample size calculation	Based on primary outcome only	Power to detect non-inferiority for primary outcome and also non-inferiority or superiority for secondary outcomes
**Analysis and reporting **		
Revising the margin for analysis	Not revised from trial design stage	Revise non-inferiority margin value based on magnitude of other benefits observed, blinded to efficacy results
Analysis and interpretation without using a margin	Typically not applicable	Decision analysis approach to give one numerical outcome reflecting efficacy, benefits, and harms, then compared between arms, or panel of clinicians and patients to make judgment of superior risk-benefit based on results
Estimands	Estimate treatment effect if all participants complete allocated treatment (hypothetical estimand; eg, impute outcomes after non-adherence)	Estimate treatment effect based on observed outcome regardless of any treatment changes (treatment policy estimand; eg, intention to treat analysis) Record treatment changes as secondary outcome
Reporting	Following CONSORT 2010 statement on non-inferiority trials	Suggest expanding CONSORT statement to include: choice of outcomes and presentation of results; updating non-inferiority margin; analysis without a margin

CONSORT= Consolidated Standards of Reporting Trials.

### Alternative objective

An alternative option non-inferiority trial aims to show that the new treatment is another option for clinicians managing their patients, possibly a more appropriate objective when multiple treatments are already in use. This objective aligns with the view of regulators, who will license new treatments based on a comparison of the efficacy of a new intervention with the current treatment, with an agreed non-inferiority margin and a safety analysis based on adverse event reporting. An example is the ALTAR (Alternatives to prophylactic antibiotics for the treatment of recurrent urinary tract infection in women) trial, investigating the role of methenamine hippurate as an alternative to antibiotic prophylaxis for urinary tract infections to reduce antimicrobial resistance at the individual and population levels.^3^ This approach, however, fails to distinguish between a non-inferior new intervention with minimal additional benefits compared with an intervention with substantial additional benefits.

### Replacement objective

A replacement non-inferiority trial aims to show that a new treatment is better than the current treatment when efficacy is evaluated together with other benefits in a risk-benefit analysis. This approach might be more appropriate for trials where the new treatment offers substantial benefits over current treatments (eg, the STREAM (The evaluation of a standardised treatment regimen of anti-tuberculosis drugs for patients with multi-drug-resistant tuberculosis) trial investigating a markedly shortened treatment regimen for drug resistant tuberculosis).^4^ As we show in our examples, a replacement non-inferiority trial might also aim to show that a new treatment is an alternative option if the results are not sufficiently favourable to view the new treatment as a replacement for the current treatment.

## Different objectives: design considerations

### Choosing non-inferiority margin

**Fig 1:**
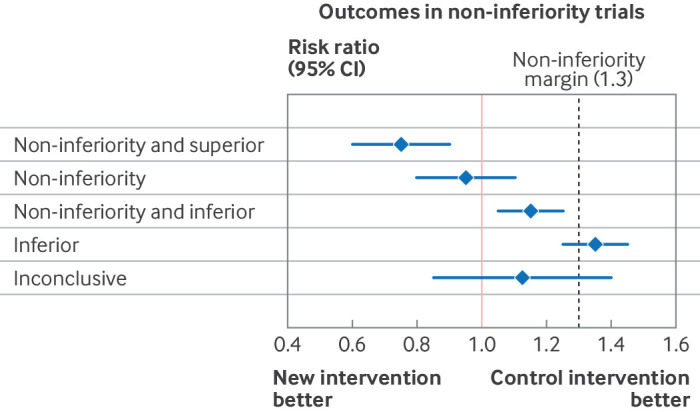
Illustration of non-inferiority based on difference in treatment effect on the risk ratio scale comparing a new intervention with an active control. Point estimates are shown with 95% confidence intervals (CI)

Conventionally, a non-inferiority trial aims to show that a new treatment is unlikely to be worse than the control arm by a defined margin based on a predefined effect measure (eg, absolute difference, relative risk, or hazard ratio).^5^ The difference in efficacy for the experimental relative to the control arm is calculated, and a maximum allowed value for the upper limit (assuming a negative outcome, such as death) or lower limit (assuming a positive outcome, such as disease remission) of the confidence interval is agreed at the design stage of the trial (non-inferiority margin; figure 1).

The non-inferiority margin should be derived differently depending on the trial objective. For alternative option trials, the margin is conventionally based on efficacy (with a safety analysis considered separately) and defined as the smallest clinically important difference. This approach is typical in licensing trials.^6^ ^7^ The value of the margin might be based on expert opinion and, especially in licensing trials, on maintenance of the effect based on evidence synthesis from previous trials that compared the current treatment with placebo.

**Fig 2:**
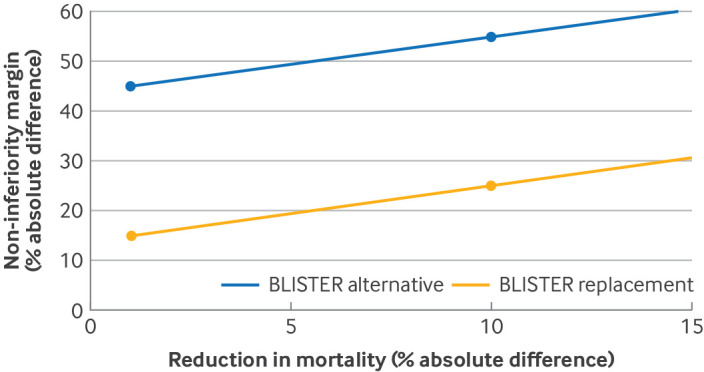
Example of hypothetical risk-benefit frontier. The Bullous Pemphigoid Steroids and Tetracyclines (BLISTER) trial proposed using different non-inferiority margins (% absolute difference) depending on the observed reduction in mortality (% absolute difference) in the new treatment arm compared with the control arm (with a greater difference allowing a wider margin). Margin values were obtained at two values of the difference in mortality (circles, one for declaration of the new intervention as an alternative option and one for replacement). Lines between the two points were plotted to show a hypothetical frontier of proposed margins at intermediate values for difference in mortality, illustrating how a more granular range for the non-inferiority margin could be used. Each measured value for the additional benefit could be associated with a non-inferiority margin, or with two non-inferiority margins, by agreeing values for an alternative option and for a replacement, as shown here, and plotting the curve. Data from Williams et al^8^

For a replacement trial, the non-inferiority margin would be based mainly on the expected magnitude of other benefits that the new treatment offers because the margin reflects the loss in effectiveness that balances the benefits (fig 2).^8^ Two approaches are possible to represent the expected other benefits on the scale of the primary outcome. A formal decision analysis approach could be undertaken to quantify the expected other benefits and potential harms relative to the primary outcome.^9^ ^10^ Alternatively, a panel of clinicians or patients, or both, could make a judgment (by reaching a consensus or by obtaining individuals’ opinions and synthesising these statistically).^11^ Ideally, guideline or policy bodies would be engaged in the selection of the margin, because ultimately their opinion is most relevant. Clearly, however, quantifying or judging the expected other benefits relative to the primary outcome will often be challenging, and might not be feasible. Also, formal decision analysis is likely to need expert support.

A proposed value for the non-inferiority margin based on one of these two methods could then be agreed and used for the trial design. If the process is carefully prespecified, however, the margin might be re-evaluated based on the other benefits seen in the trial, or the final analysis might be conducted without reference to a margin (discussed in Different objectives: analysis and reporting).

### Choice of outcomes

The primary outcome, chosen to measure the efficacy of the treatment, will typically be an established measure for the disease being treated, such as survival, similar to a superiority trial. In an alternative option trial, secondary outcomes that measure harms and benefits would not be considered as important as the primary outcome unless a safety signal of concern was present. In replacement trials, secondary outcomes should comprehensively reflect the important benefits and harms of the treatments and might even be considered joint primary outcomes.

The selection of benefits and risks to be assessed as outcomes in a trial should be chosen carefully. Regulatory bodies often have criteria about who should be consulted for opinions about acceptable risk. Geographic variation in prioritising risks and benefits exists, and the availability of resources will affect this selection. Guideline bodies should also be consulted to ensure that information they regard as important is collected.

### Sample size calculations

A wider non-inferiority margin gives a less restrictive definition of non-inferiority and hence typically means smaller sample sizes; we already discussed how the non-inferiority margin is influenced by the trial objective. Consideration of the precision of estimates for secondary outcomes will also be important for replacement trials but not necessarily for alternative option trials. A replacement trial could even be powered based on non-inferiority in efficacy and superiority in outcomes that measure the other benefits (with separate calculations and the largest sample size value used, or more formally considering power to jointly show non-inferiority and superiority). Alternatively, a decision theory approach to sample size calculation could be taken for a replacement trial, to account for both efficacy and other benefits jointly.^12^

### Estimands

The recently published ICH (International Council for Harmonisation of Technical Requirements for Pharmaceuticals for Human Use) addendum E9 (R1) described strategies for handling events occurring after a clinical trial participant is randomised that would affect the interpretation of the outcome (known as intercurrent events).^13^ These strategies are part of the definition of estimands, and are intended to improve the interpretability and clinical relevance of treatment effects in clinical trials. A key intercurrent event in many non-inferiority trials is changes in treatment, and these should always be reported clearly. A hypothetical estimand compares the outcomes if the patient had not changed treatment: it represents the efficacy of the participant’s initially allocated treatment. Conversely, a treatment policy estimand involves the actual outcome for a participant, irrespective of whether the treatment has changed during the trial, and aims to determine the real world effectiveness of the treatment as part of a treatment policy, including second line treatment options.

An alternative option trial could use the hypothetical estimand to assess maintenance of effect and compare only the efficacy of treatment. For a replacement trial, the treatment policy estimand would be more relevant for comparing treatments in a risk-benefit framework, because these comparisons are typically more focused on pragmatic questions. Levels of switching treatment should be recorded as a secondary outcome, however, and high levels of switching typically mean a reduction in the additional benefits of a new intervention, which should be reflected in the analysis. In some contexts, high levels of switching from a new treatment will question its use, but in others, avoiding an intensive or expensive current treatment, such as surgery, in even a small number of patients, will be an important benefit.

## Different objectives: analysis and reporting

### Revising the non-inferiority margin for analysis

Typically, the non-inferiority margin is not updated during alternative option trials, because these trials are designed to assess maintenance of efficacy that would not generally be influenced by other events in the trial. For example, safety signals do not lead to a modification of the margin, but rather to an experimental arm being discontinued or the trial stopped.^14^ Two possible reasons to change the margin, however, are non-constancy of effect^15^ and if the risk in the control arm is different from that assumed at study design.^16^

For a replacement trial, the non-inferiority margin at study design would be based on the assumed magnitude of the other benefits. Because the magnitude of the other benefits is investigated by collecting secondary outcomes during the trial, revising the margin before conducting the final analysis of the primary outcome is logical.^15^ This procedure is not commonly done at present. The procedure would require steps to ensure that the analysis of the secondary outcomes was conducted without access to the primary outcome data,^17^ with subsequent consideration of how the non-inferiority margin might consequently be modified. One approach could be to define the relation between the level of other benefits observed and the margin before the start of the trial. If there is one key benefit of the new treatment, or if the benefits can be summarised as a score, then the relation could be plotted in a graph similar to that in figure 2. In figure 2, a potential relation is illustrated for the BLISTER (Bullous Pemphigoid Steroids and Tetracyclines) trial.^8^ Whereas the original trial used two different values for the non-inferiority margin (for the alternative option or the replacement) at each of two values of the additional benefit (reduction in mortality), the graph shows how the value for the non-inferiority margin could be specified across a range of measured values for the prespecified additional benefit. This approach mimics the non-inferiority frontier method to modify the non-inferiority margin based on the risk in the control arm found in a trial,^16^ which could potentially be adapted to respond to the magnitude of other benefits seen in the trial.

### Analysis and interpretation without using a margin

To compare the risk-benefit profile between arms in replacement trials, a decision analysis approach can be used to assign one numerical outcome value (a utility) to each participant that represents the overall balance of benefit and harm,^18^ with benefits typically assigned positive numbers and harms negative numbers. Such a system could score, for example, +1 for a successful cure and defined benefits, −1 for extension of treatment, and −1 to −10 for adverse events of varying severity. Bayesian approaches could incorporate uncertainty in the relative values of different benefits and harms. An alternative is for the results of key outcomes representing harms and benefits to be presented to an independent panel of clinicians or patients, or both, for them to decide if the new treatment is superior in terms of overall risk-benefit.^19^ This method would correspond to the approach taken by many treatment recommendation bodies.

### Reporting

The Consolidated Standards of Reporting Trials (CONSORT) 2010 statement has been extended for non-inferiority trials.^20^ The checklist includes most key items relevant for non-inferiority trials in general, but seems to have been developed only for alternative option trials. Specifically, items 4a, 5, and 6a require comparison of the eligibility criteria, nature of the current treatment, and outcomes in the non-inferiority trial with those in the trial or trials that have established the efficacy of the current treatment (relative to placebo). These three items are typically not relevant for a replacement trial. Further items not currently listed explicitly but which are important to understand, particularly for a replacement trial, are choice of outcomes and presentation of results for other benefits of the new treatment, whether the non-inferiority margin used for design is updated for analysis based on this or other information, and whether a margin is used for analysis.

## Replacement objective: contrast with a superiority trial

A risk-benefit superiority trial design could be used for trials trying to show that a new therapeutic agent should replace standard of care. This design defines a primary outcome that measures the risk-benefit profile, such as an existing quality of life score or a bespoke scoring system. This outcome is equivalent to the overall utility score that can be used for analysis of a non-inferiority trial without using a margin.^10^ ^12^

Arguments exist for and against the superiority and non-inferiority approaches when comparing treatments in terms of risk-benefit,^21^ and their relative merits deserve more research. For example, a superiority trial might be considered simpler conceptually and could potentially require a smaller sample size. This design would also capture the risks and benefits in the same people because they are reflected in the primary outcome for each individual.^22^ In contrast, when consulting a panel to interpret findings for harms and benefits reported separately in a non-inferiority trial, the problem of how harms and benefits overlap in the same individual is typically ignored. The non-inferiority design for a trial could nevertheless allow for more individualised thinking about the balance between benefits and harms by including separate harm and benefit outcomes (although these can also be reported in a superiority trial), and therefore avoids over-reliance on how trade-offs were judged by those experts or patients consulted by the trialists. A non-inferiority trial would also show that a treatment was an alternative option, should the additional benefits be less than expected at the design of the trial, for example by specifying separate margins for the two different objectives, as shown in our examples.^8^ ^23^

## Adopting alternative option and replacement non-inferiority trials

Table 2 describes the main considerations for adopting alternative option and replacement non-inferiority trials. The appropriateness of using the alternative option and replacement trial models will depend on patient and disease factors, the nature of the additional benefits of the new intervention, and whether different aspects of treatment response can be reliably measured.

**Table 2:** Considerations in the choice of non-inferiority trial objective based on patient, disease, and other characteristics

Criteria	Acceptable alternative	Replacement
No of standard of care treatments	Numerous (because of numerous comparators; trial including all is not feasible)	One standard of care
Magnitude of additional benefits of new intervention	Modest	Substantial
Measurement of additional benefits of new intervention	Challenging to accurately measure	Possible to accurately measure, or fixed and inherent in intervention (eg, shorter duration)
Ability to trade-off efficacy and other benefits	Challenging as little consensus on how additional benefits can be expressed on scale of primary (efficacy) outcome	Can be traded off through established (eg, quality of life score) or newly developed decision analysis tool, or a body of experts and patients, or guideline or policy makers, can be convened to give judgment on trade-off

Criteria are not prescriptive, but each should be considered before designing a trial. A replacement trial can be considered when all of the criteria are met.

In general, the alternative strategy would be a more appropriate design for a trial investigating a new intervention when multiple treatment options are already available for the disease or condition and the magnitude of the additional benefits is predicted to be modest. This approach is also recommended if the additional benefits cannot be accurately described or attributed to the intervention (eg, feeling well enough to return to work). In this context, determining how the additional benefits could be expressed on a suitable scale to be formally compared between treatments would be challenging. 

The replacement trial design would be suitable for use if a common standard of care is applied for most patients, but a new intervention is expected to have substantial additional benefits. If these additional benefits are fixed (eg, a much shorter duration), can be accurately determined (eg, a substantially improved toxicity profile), or both, the new intervention can be compared with the standard of care with the intention of becoming a replacement. Efficacy can be assessed, and the magnitude of the additional benefits determined for the experimental and standard of care arms.

## Examples of replacement non-inferiority trials

We present two example trials which show how the risk-benefit profile can be used in the non-inferiority setting. Although the trials were designed with the replacement objective as the ultimate goal, the less ambitious alternative option objective was also considered within these trials. Analysis can be considered to sequentially test statistically whether the new treatment is a replacement and, if not, then testing whether it is an alternative option.

### Medical Research Council randomised trial in testicular teratoma

In this trial, patients were randomised to receive bleomycin and etoposide with either cisplatin (BEP, the existing treatment) or carboplatin (BEC, the experimental treatment).^23^ Recurrence free survival and overall survival were defined as outcomes of the efficacy of the treatments, and toxic effects indicated other benefits of BEC (table 3). Based on our terminology, and because of the reduction in toxic effects expected at the trial design, BEC would be considered a replacement if the absolute difference in reduction in recurrence free survival was <10%, BEP would be recommended if this value was >15%, and the recommendation would be uncertain if the reduction was between 10% and 15% (BEC would be an alternative option). Although the difference scale is used in both examples, relative scales might be more robust to a lower or higher event rate than expected in the control arm. 

**Table 3:** Design considerations with real world examples: two trials that incorporated the alternative option and replacement objectives in their design with different methods

Trial characteristics	MRC teratoma trial	BLISTER trial
Disease area	Testicular teratoma	Bullous pemphigoid
Outcomes	Efficacy: overall survival (primary), relapse free survival Additional benefits: adverse events	Efficacy: ≤3 blisters at six weeks Additional benefits: adverse events, mortality
Control arm	Bleomycin-etoposide-cisplatin	Steroids
Experimental arm	Bleomycin-etoposide-carboplatin	Doxycycline
Alternative option non-inferiority margin (absolute difference)	15%	45%, assuming an absolute mortality reduction of 1% or 55%, assuming an absolute mortality reduction of 10%
Replacement non-inferiority margin (absolute difference)	10%	15%, assuming an absolute mortality reduction of 1% or 25%, assuming an absolute mortality reduction of 10%

The Medical Research Council (MRC) randomised trial in testicular teratoma (which was stopped early) proposed two non-inferiority margins with the intention of declaring the new intervention as a replacement if the confidence interval for the difference between the two treatment arms was less than the smaller of the margins, both margins chosen based on an expected reduction in adverse events of the new treatment. The BLISTER (Bullous Pemphigoid Steroids and Tetracyclines) trial also proposed two margins, but these were chosen (prespecified) based on two different potential reductions in mortality by the new treatment because of the uncertainty of the magnitude of the reduction in mortality.

While the trial was running, ondansetron was introduced and the side effects of BEP were better managed. Also, evidence emerged suggesting that relapses associated with BEC were more difficult to manage. Therefore, clinicians collaborating with the data safety monitoring committee reported at an interim analysis that their personal non-inferiority margin was reduced. This finding suggested revising the margin to a smaller size (and increasing the sample size). The trial was stopped, however, because of unfavourable results for BEC.

This example illustrates how trialists can select a non-inferiority margin explicitly to facilitate a treatment recommendation at the end of the trial, how this margin can be appropriately revised during the trial, and how this could have been reviewed again at the end of the trial before the final analysis. A formal decision analysis method or opinions from a panel could have investigated the balance between benefits and risks, in particular about the need for an additional drug treatment (ondansetron).

### BLISTER trial

The BLISTER trial investigated whether doxycycline was non-inferior to steroid treatment (standard of care) when treating bullous pemphigoid, with the primary efficacy outcome of the presence of ≤3 blisters at six weeks.^8^ Safety was assessed based on adverse events related to treatment, including death. The expectation was that doxycycline (a tetracycline antibiotic) would be less efficacious, but less toxic than steroid treatment.

Non-inferiority margins were obtained from a panel of clinicians from two hypothetical scenarios for the reduction in mortality because of the uncertainty of the magnitude of the reduction (table 3). Hypothetical differences in mortality of 1% or 10% less for doxycycline were assumed and clinicians decided which non-inferiority margins would be used to consider the treatment as an alternative option or replacement, respectively (supplementary material in Williams et al^8^). The margins indicated that a modest reduction in mortality was considered desirable, even with a large reduction in efficacy, particularly because patients who did not respond to doxycycline could subsequently be offered steroids.

Ultimately, a pragmatic non-inferiority margin of 37% was chosen at study design for the primary efficacy outcome and the sample size was based on an assumed 25% reduction in efficacy in the doxycycline arm. The results showed that the efficacy of the primary outcome in the doxycycline arm was worse than steroids by 18.6% (90% confidence interval 11.1% to 26.1%), but adverse events were less (19.0%, 7.9% to 30.1%). Doxycycline was declared non-inferior based on the non-inferiority margin at study design. The trialists noted that the upper bound of the 95% confidence interval for efficacy (26.1%) was close to the 25% margin that could be traded off against a larger reduction in adverse events (specifically mortality) to recommend doxycycline as a replacement for steroids (as first line treatment). However, the trialists did not directly make this recommendation. In principle, the trialists could have presented the panel of clinicians with the adverse events results and got new margins for the final efficacy analysis.

## Concluding remarks

We have presented the steps required to clarify the intention when conducting a non-inferiority trial. We have argued that important differences exist between trials that compare treatments for efficacy only and those that assess overall risk-benefit, and thus aim to show the new intervention is an additional option or a replacement for standard of care, respectively. We have provided guidance on how to choose the correct objective for a trial (table 2), and how this choice should influence the design, analysis, and reporting of the trial (table 1). Box 1 summarises our recommendations for practice and highlights key areas for further research and consideration on how best to implement our recommendations, taking into account the needs of stakeholders. Designers of clinical trials are in an ideal position to collect data about the additional benefits of a new intervention and present these findings in a statistically appropriate way: when appropriate, the replacement approach to design and analysis would help individual healthcare providers and policy makers, and could promote wider uptake and more benefit to patients in a shorter time.

Box 1 **Summary of recommendations for practice for non-inferiority trials, and suggested guidance for next steps to fully establish the two objectives approach**

**Key recommendations for practice**

- Investigators should decide before designing a non-inferiority trial whether the objective is to show that a new intervention is an alternative option or a replacement for standard of care.
- Key considerations include the magnitude of the additional benefits of the new intervention and the balance between these additional benefits and possible loss of efficacy.
- Trial design, analysis, and reporting should reflect the objective chosen; the objective influences how systematically and extensively additional benefits or harms of treatment are collected, and the non-inferiority margin.
- To show that a new treatment is a replacement for standard of care, decision analysis is recommended or judgment by a panel of clinicians or patients, based on efficacy results, additional benefits, and other relevant secondary outcomes.
- Trialists might conduct a decision analysis or convene a panel themselves, or intentionally collect sufficient data for policy makers or funders, to determine that a new treatment is a replacement for standard of care.

**Guidance for next steps**

- More methodological research is needed on how best to define, and possibly redefine, a non-inferiority margin based on the magnitude of additional benefits expected or found in a trial.
- Protocol templates and funding bodies should acknowledge the value of an active decision to measure additional benefits in an adequately powered way.
- The clinical trial community should engage regulatory bodies in discussion about how best to incorporate the two objectives in regulatory trials.
- More research is needed to identify the priorities of stakeholder about the two objectives in different disease areas, and the implications for design and analysis of trials.

## Notes

Contributors: All authors are part of the methodology theme at the Medical Research Council (MRC) Clinical Trials Unit at University College London with experience in non-inferiority trial methodology relating to design and analysis. The main sources used in the development of the article are included as references in the text. AJC and CDT developed the original version of the manuscript, and accompanying figures and tables. MNC, RMT, AJN, DTD, and IRW provided input on the methodology and considerations presented here, and suggestions on developing the manuscript. All authors commented on each draft of the manuscript, with substantial changes made as a result. All authors also approved the final version. AJC is the guarantor. The corresponding author attests that all listed authors meet authorship criteria and that no others meeting the criteria have been omitted.

Provenance and peer review: Not commissioned; externally peer reviewed.
